# A 10-year retrospective cross-sectional study of severe cutaneous adverse drug reactions in Thailand: Human leukocyte antigen typing and enzyme-linked immunospot assay-assisted drug identification

**DOI:** 10.1016/j.jdin.2025.10.013

**Published:** 2025-11-01

**Authors:** Chanidapa Wongtada, Kridipop Charoenchaipiyakul, Pajaree Chariyavilaskul, Yuda Chongpison, Supranee Buranapraditkun, Pattarawat Thantiworasit, Pungjai Mongkolpathumrat, Leena Chularojanamontri, Papapit Tuchinda, Yuttana Srinoulprasert, Ticha Rerkpattanapipat, Kumutnart Chanprapaph, Wareeporn Disphanurat, Panlop Chakkavittumrong, Padcha Pongcharoen, Napatra Tovanabutra, Chutika Srisuttiyakorn, Chonlaphat Sukasem, Kanokkarn Pinyopornpanish, Pawinee Rerknimitr, Jettanong Klaewsongkram

**Affiliations:** a Division of Dermatology, Department of Medicine, Faculty of Medicine, Center of Excellence for Skin and Allergy Research (CESAR), Chulalongkorn University, Bangkok, Thailand; b Department of Pharmacology, Faculty of Medicine, Chulalongkorn University, Bangkok, Thailand; c Center of Excellence in Clinical Pharmacokinetics and Pharmacogenomics, Department of Pharmacology, Faculty of Medicine, Chulalongkorn University, Bangkok, Thailand; d Biostatistics Excellence Center, Research Affairs Division, Faculty of Medicine, Center of Excellence for Skin and Allergy Research (CESAR), Chulalongkorn University, Bangkok, Thailand; e Division of Allergy and Clinical Immunology, Department of Medicine, Faculty of Medicine, Center of Excellence for Skin and Allergy Research (CESAR), Chulalongkorn University, Bangkok, Thailand; f Department of Dermatology, Faculty of Medicine Siriraj Hospital, Mahidol University, Bangkok, Thailand; g Department of Immunology, Faculty of Medicine Siriraj Hospital, Mahidol University, Bangkok, Thailand; h Division of Allergy, Immunology and Rheumatology, Department of Medicine, Faculty of Medicine, Ramathibodi Hospital, Mahidol University, Bangkok, Thailand; i Division of Dermatology, Department of Medicine, Faculty of Medicine, Ramathibodi Hospital, Mahidol University, Bangkok, Thailand; j Division of Dermatology, Department of Medicine, Faculty of Medicine, Thammasat University, Pathumthani, Thailand; k Division of Dermatology, Department of Internal Medicine, Chiang Mai University, Chiang Mai, Thailand; l Division of Dermatology, Department of Medicine, Phramongkutklao Hospital, Phramongkutklao College of Medicine, Bangkok, Thailand; m Division of Pharmacogenomics and Personalized Medicine, Department of Pathology, Faculty of Medicine, Ramathibodi Hospital, Mahidol University, Bangkok, Thailand; n Pharmacogenomics and Precision Medicine Clinic, Bumrungrad Genomic Medicine Institute, Bumrungrad International Hospital, Bangkok, Thailand; o Division of Allergy and Clinical Immunology, Department of Internal Medicine, Faculty of Medicine, Chiangmai University, Chiangmai, Thailand

**Keywords:** AGEP, DRESS, drug hypersensitivity, GBFDE, HLA-B, IFN-γ ELISpot, severe cutaneous adverse drug reactions, SJS/TEN, Thailand

*To the Editor:* Severe cutaneous adverse drug reactions (SCARs), including Stevens-Johnson syndrome/toxic epidermal necrolysis (SJS/TEN), drug reaction with eosinophilia and systemic symptoms (DRESS), acute generalized exanthematous pustulosis (AGEP), and generalized bullous fixed drug eruption (GBFDE), are dermatological emergencies with significant morbidity and mortality.^1^ This 10-year retrospective cross-sectional study was conducted based on data from the ThaiSCAR project, a registry of SCARs from 6 tertiary centers in Thailand (2013-2022), to delineate clinical patterns, culprit drugs, human leukocyte antigen (HLA)-B associations, and the performance of interferon-γ enzyme-linked immunospot (ELISpot) assays in determining the culprit drugs. For details of the methods and results, see Supplementary Fig 1, available via Mendeley at https://data.mendeley.com/datasets/htyt6mnkn6/1 and Tables I-VII, available via Mendeley at https://data.mendeley.com/datasets/htyt6mnkn6/1.

A total of 480 patients with probable or definite SCARs (registry of severe cutaneous adverse reactions/European study of severe cutaneous adverse reactions criteria) were included.^2^, ^3^, ^4^ SJS/TEN predominated (46.3%), followed by DRESS (35.6%), AGEP (16.0%), and GBFDE (2.1%). Median age was 55 years, with AGEP patients significantly older at 69 years (Table I). Overall SCAR incidence peaked in December (11.4%), while AGEP cases were significantly high in September (17.1%) and November (14.5%). Supplementary Fig 1, available via Mendeley at https://data.mendeley.com/datasets/htyt6mnkn6/1 Clinical features, culprit drugs, and latency varied by SCAR phenotypes. (Table I, Supplementary Tables I and II, available via Mendeley at https://data.mendeley.com/datasets/htyt6mnkn6/1) Anticonvulsants (16.5%) and beta-lactams (18.7%) were leading culprits. Anticonvulsants predominated in SJS/TEN and DRESS; allopurinol, sulfonamides, antituberculosis, and antiretroviral drugs were more frequent in DRESS; beta-lactams in AGEP, and nonsteroidal anti-inflammatory drugs/analgesics in GBFDE. There were 14.6% of the patients with HIV infection, who were mostly SJS/TEN (58.6%) or DRESS (40.0%). The latency period in HIV patients with DRESS was significantly longer than non-HIV patients.(Supplementary Table III, available via Mendeley at https://data.mendeley.com/datasets/htyt6mnkn6/1)

**Table I:** Demographic and clinical characteristics of SCAR patients

Demographic data	Total	SJS/TEN				DRESS	AGEP	GBFDE	*P* value
		All SJS/TEN	SJS	SJS/TEN	TEN				
No. of cases, *N* (%)	480 (100.0)	222 (46.3)	146 (30.4)	35 (7.3)	41 (8.5)	171 (35.6)	77 (16.0)	10 (2.1)	
Age (y)									<.001∗
Median (Q1, Q3)	55.0 (37.5, 69.0)	51.0 (33.0, 65.0)	50.5 (33.0, 64.0)	45.0 (30.0,62.0)	56.0 (47.0,68.0)	55.0 (39.0, 68.0)	69.0 (57.0, 80.0)†	52.5 (30.0, 65.0)	
Sex, *N* (%)									.004∗
Male	214 (44.6)	104 (46.9)	67 (45.9)	19 (54.3)	18 (43.9)	84 (49.1)	19 (24.7)†	7 (70.0)	
Female	266 (55.4)	118 (53.1)	79 (54.1)	16 (45.7)	23 (56.1)	87 (50.9)	58 (75.3)†	3 (30.0)	
Race and ethnicity, *N* (%)									.196
Thai	467 (97.3)	214 (96.4)	143 (97.9)	31 (88.6)	40 (97.6)	167 (97.7)	77 (100.0)	9 (90.0)	
Others‖	13 (2.7)	8 (3.7)	3 (2.1)	4 (11.4)	1 (2.4)	4 (2.3)	0 (0.0)	1 (10.0)	
Underlying diseases, *N* (%)									
Atopy	22 (4.6)	15 (6.8)	12 (8.2)	2 (5.7)	1 (2.4)	4 (2.3)	3 (3.9)	0 (0.0)	.177
Liver disease	31 (6.5)	17 (7.7)	12 (8.2)	4 (11.4)	1 (2.4)	11 (6.4)	3 (3.9)	0 (0.0)	.561
Renal insufficiency	79 (16.5)	32 (14.4)	20 (13.7)	4 (11.4)	8 (19.5)	26 (15.2)	19 (24.7)	2 (20.0)	.192
Autoimmune/rheumatic diseases	52 (10.8)	25 (11.4)	23 (15.8)	2 (5.7)	0 (0.0)	15 (8.8)	12 (15.6)	0 (0.0)	.283
Diabetes mellitus type II	91 (19.0)	34 (15.3)	19 (13.0)	9 (25.7)	6 (14.6)	26 (15.2)	30 (39.0)†	1 (10.0)	<.001∗
Hypertension	133 (27.7)	57 (25.7)	35 (24.0)	9 (25.7)	13 (31.7)	37 (21.6)	37 (48.1)†	2 (20.0)	<.001∗
Dyslipidemia	100 (20.8)	36 (16.2)	22 (15.1)	6 (17.1)	8 (19.5)	31 (18.1)	32 (41.6)†	1 (10.0)	<.001∗
Neurological diseases	126 (26.3)	54 (24.3)	35 (24.0)	6 (17.1)	13 (31.7)	48 (28.1)	24 (31.2)	0 (0.0)	.155
Malignancy	79 (16.5)	40 (18.0)	28 (19.2)	5 (14.3)	7 (17.1)	23 (13.5)	13 (16.9)	3 (30.0)	.413
HIV infection	70 (14.6)	41 (18.5)	28 (19.2)	11 (31.4)	2 (4.9)	28 (16.4)	1 (1.3)†	0 (0.0)	.001∗
Cardiovascular disease	81 (16.9)	30 (13.5)	17 (11.6)	3 (8.6)	10 (24.4)	26 (15.2)	24 (31.2)†	1 (10.0)	.003∗
Pulmonary diseases	74 (15.4)	31 (14.0)	24 (16.4)	6 (17.1)	1 (2.4)	34 (19.9)	8 (10.4)	1 (10.0)	.196
History of drug allergy, *N* (%)	91 (19.0)	38 (17.1)	23 (15.8)	5 (14.3)	10 (24.4)	26 (15.2)	24 (31.2)†	3 (30.0)	.016∗
SCARs	19 (4.0)	8 (3.6)	3 (2.1)	3 (8.6)	2 (4.9)	5 (3.0)	6 (7.8)	0 (0.0)	.268
Clinical characteristics									
Culprit drugs, *N* (%)	(N = 715)	(*N* = 324)	*N* = 214	*N* = 54	*N* = 56	*N* = 237	*N* = 134	*N* = 20	
Anticonvulsants	118 (16.5)	67 (20.7)	46 (21.5)	6 (11.1)	15 (26.8)	49 (20.7)	2 (1.5)†	0 (0.0)	<.001∗
Aromatic	98 (13.7)	56 (17.3)	41 (19.2)	6 (11.1)	9 (16.1)	41 (17.3)	1 (0.7)	0 (0.0)	.404
Nonaromatic	20 (2.8)	11 (3.4)	5 (2.3)	0 (0.0)	6 (10.7)	8 (3.4)	1 (0.7)	0 (0.0)	<.001∗
Allopurinol	76 (10.6)	34 (10.5)	21 (9.8)	5 (9.3)	8 (14.3)	41 (17.3)	1 (0.7)	0 (0.0)	<.001∗
Beta-lactams	134 (18.7)	43 (13.3)	24 (11.2)	7 (13.0)	12 (21.4)	19 (8.0)	68 (50.8)†	4 (20.0)	<.001∗
Sulfonamides/sulfones	87 (12.2)	41 (12.7)	33 (15.4)	6 (11.1)	2 (3.6)	41 (17.3)	2 (1.5)†	3 (15.0)	.029∗
Anti-TB	48 (6.7)	20 (6.2)	11 (5.1)	8 (14.8)	1 (1.8)	24 (10.1)‡	4 (3.0)	0 (0.0)	.016∗
Other antimicrobials¶	139 (19.4)	67 (20.7)	41 (19.2)	16 (29.6)	10 (17.9)	32 (13.5)†	36 (26.9)	4 (20.0)	<.001∗
Other antibiotics	96 (13.4)	45 (13.9)	30 (14.0)	8 (14.8)	7 (12.5)	14 (5.9)†	33 (24.6)§	4 (20.0)	.260
Vancomycin	24 (3.4)	9 (2.8)	7 (3.3)	0 (0.0)	2 (3.6)	5 (2.1)	9 (6.7)	1 (4.8)	.059
Antifungal	20 (2.8)	15 (4.6)	6 (2.8)	6 (11.1)	3 (5.4)	3 (1.3)	2 (1.5)	0 (0.0)	.003∗
ARV	22 (3.1)	7 (2.2)	5 (2.3)	2 (3.7)	0 (0.0)	15 (6.3)†	0 (0.0)	0 (0.0)	<.001∗
NSAIDs/analgesics	49 (6.9)	27 (8.3)	22 (10.3)	2 (3.7)	3 (5.4)	7 (3.0)	6 (4.5)	9 (45.0)†	.272
Others	64 (8.9)	25 (7.7)	16 (7.5)	4 (7.4)	5 (8.9)	24 (10.1)	15 (11.2)	0 (0.0)	
Latency peroid (d, median (Q1, Q3))	(*N* = 693) 13 (5,25)	(*N* = 315) 13 (8, 22)	(*N* = 210) 13 (8, 21)	(*N* = 51) 13 (8, 30)	(*N* = 54) 13 (9, 17)	(*N* = 229) 25 (13, 37)	(*N* = 129) 3 (2, 6)	(*N* = 20) 1 (1, 4)	<.001∗
Duration of admission (d, median (Q1, Q3))	(*N* = 400) 10 (6, 24)	(*N* = 181) 10 (6, 24)	(*N* = 118) 8.5 (6, 17)	(*N* = 29) 15 (6, 32)	(*N* = 34) 22.5 (12, 50)	(*N* = 141) 9 (5, 18)	(*N* = 71) 16 (6, 38)	(*N* = 7) 26 (4, 42)	.035∗
Rash onset, *N* (%)	(*N* = 468)	(*N* = 218)	(*N* = 145)	(*N* = 35)	(*N* = 38)	(*N* = 164)	(*N* = 77)	(*N* = 9)	<.001∗
Prior to hospitalization	346 (73.9)	177 (81.2)	121 (83.5)	26 (74.3)	30 (78.9)	132 (80.5)	32 (41.6)†	5 (55.6)	
During hospitalization	122 (26.1)	41 (18.8)	24 (16.6)	9 (25.7)	8 (21.1)	32 (19.5)	45 (58.4)†	4 (44.4)	
In-hospital death, *N* (%)	39 (8.9) (*N* = 438)	27 (13.1)† (*N* = 206)	13 (9.3) (*N* = 140)	2 (6.3) (*N* = 32)	12 (35.3) (*N* = 34)	5 (3.4) (*n* = 148)	7 (9.3) (*N* = 75)	0 (0.0) (*N* = 9)	<.001∗

*AGEP*, Acute generalized exanthematous pustulosis; *ARV*, antiretroviral drugs; *DRESS*, drug reaction with eosinophilia and systemic symptoms; *GBFDE*, generalized bullous fixed drug eruption; *NSAID*, nonsteroidal anti-inflammatory drug; *SCAR*, severe cutaneous adverse drug reaction; *SJS/TEN*, Stevens–Johnson syndrome/toxic epidermal necrolysis; *TB*, tuberculosis.

∗ *P* < .05 among all SCAR phenotypes.

† *P* < .05 compared to other SCAR phenotypes.

‡ *P* < .05 compared to AGEP.

§ *P* < .05 compared to SJS/TEN and DRESS.

‖ Including Myanmar, Chinese, and Indian.

¶ Other antimicrobials include ARV (tenofovir, lamivudine, efavirenz, emtricitabine, nevirapine, dolutegravir), antifungal drugs (fluconazole, amphotericin, terbinafine, flucytosine), and other antibiotics (clindamycin, macrolide, fluoroquinolones, colistin, tetracyclines, fosfomycin, linezolid, metronidazole, vancomycin, aminoglycoside).

Interferon-γ ELISpot (340 patients, 516 drugs) showed 39.7% overall positivity, highest in DRESS (52.5%), followed by SJS/TEN (38.6%), AGEP (23.9%), while GBFDE was consistently negative. Anticonvulsants showed the highest positivity (49.4%), especially in DRESS (62.1%), whereas lower positivity was seen in nonsteroidal anti-inflammatory drugs/analgesic drugs (Table II, Supplementary Table IV, available via Mendeley at https://data.mendeley.com/datasets/htyt6mnkn6/1). Comorbidities such as diabetes, hypertension, dyslipidemia, renal insufficiency, and atopy correlated with reduced positivity. Supplementary Table V, available via Mendeley at https://data.mendeley.com/datasets/htyt6mnkn6/1 HLA-B genotyping in 382 patients revealed HLA-B∗15:02 (12.4%), HLA-B∗58:01 (12.3%), HLA-B∗46:01 (10.7%), HLA-B∗13:01 (9.7%), and HLA-B∗40:01 (7.2%) as most prevalent. Certain genotype-drug associations were confirmed: HLA-B∗15:02 with carbamazepine-induced SJS/TEN, HLA-B∗58:01 with allopurinol-induced DRESS, and HLA-B∗13:01 with sulfonamide/sulfone-induced DRESS. Carriers exposed to sulfonamide/sulfones had markedly increased risk of DRESS (adjusted odds ratio 21.313, 95% CI 5.700-79.681), while no association was observed among nonusers (adjusted odds ratio 1.777, 95% CI 0.943-3.348). Supplementary Table VI, available via Mendeley at https://data.mendeley.com/datasets/htyt6mnkn6/1 In-hospital mortality ranged from 0% in GBFDE to 35.3% in TEN. Higher mortality was associated with older age, renal insufficiency, malignancy, and cardiovascular disease. SJS/TEN cases with extensive detachment (>30%), large epidermal sheets, and genital mucosal involvement were also at higher risks.(Supplementary Table VII, vailable via Mendeley at https://data.mendeley.com/datasets/htyt6mnkn6/1)

**Table II:** IFN-γ ELISpot assay according to SCAR phenotypes and drug types

IFN-γ ELISpot responses	*N* (%) of positive IFN-γ ELISpot assay (cut off ≥20 cells/10^6^ PBMCs)	Frequencies of drug-specific IFN-γ-releasing cells/10^6^ PBMCs (median (Q1, Q3))
SCAR phenotypes (as person)		
Overall	135/340 (39.7%)	100 (48, 252)
SJS/TEN	56/145 (38.6%)	100 (46, 228)
DRESS	63/120 (52.5%)∗	112 (52, 264)
AGEP	16/67 (23.9%)	86 (44, 266)
GBFDE	0/8 (0.0%)	-
Drug types (as each drug)		
All drug types	154/516 (29.8%)	92 (44, 204)
Anticonvulsants	38/77 (49.4%)†	138 (64, 332)
Allopurinol	24/55 (43.6%)†	60 (42, 149)
Beta-lactams	22/103 (21.4%)	78 (41, 188)
Sulfonamides/sulfones	24/68 (35.3%)	114 (54, 192)
Anti-TB	17/39 (43.6%)	64 (46, 150)
Other antimicrobial drugs‡	15/103 (14.6%)	100 (32, 284)
NSAIDs/analgesic	6/29 (20.7%)	56 (40, 108)
Others	8/42 (19.1%)	50 (34, 110)

*AGEP*, Acute generalized exanthematous pustulosis; *BSA*, body surface area; *DRESS*, drug reaction with eosinophilia and systemic symptoms; *ELISpot*, enzyme-linked immunospot; *GBFDE*, generalized bullous fixed drug eruption; *NSAID*, nonsteroidal anti-inflammatory drug; *PBMC*, peripheral blood mononuclear cell; *SJS/TEN*, Stevens–Johnson syndrome/toxic epidermal necrolysis; *TB*, tuberculosis.

∗ *P* < 0.05 compared to other SCAR phenotypes.

† *P* < 0.05 compared to NSAIDs/analgesic, beta-lactams and other antimicrobial drugs.

‡ Other antimicrobials include ARV (tenofovir, lamivudine, efavirenz, emtricitabine, nevirapine, dolutegravir), antifungal drugs (fluconazole, amphotericin, terbinafine, flucytosine), and other antibiotics (clindamycin, macrolide, fluoroquinolones, colistin, tetracyclines, fosfomycin, linezolid, metronidazole, vancomycin, aminoglycoside).

This large-scale real-world cohort underscores distinct epidemiological and immunogenetic patterns of SCARs in Thailand. Although no mortality was observed in GBFDE cases in our study, a previous report documented a 22% rate, which may be attributable to older patient age and greater disease severity (>10% body surface area detachment).^4^ ELISpot is promising for culprit drug identification, though its sensitivity is variable.^5^ It appears particularly beneficial in DRESS and cases with anticonvulsants, where positivity rates are relatively high. Further studies are required to define factors affecting its performance and optimize clinical utility. HLA genotyping remains central to risk prediction. This study validated established associations, identified potential risk and protective alleles, and supported precision prescribing and safer practice.

## Conflicts of interest

None disclosed.

## References

[1] Hung S.-I., Mockenhaupt M., Blumenthal K.G. (2024). Severe cutaneous adverse reactions. Nat Rev Dis Primers.

[2] Sidoroff A., Halevy S., Bavinck J.N., Vaillant L., Roujeau J.C. (2001). Acute generalized exanthematous pustulosis (AGEP)--a clinical reaction pattern. J Cutan Pathol.

[3] Kardaun S.H., Sidoroff A., Valeyrie-Allanore L. (2007). Variability in the clinical pattern of cutaneous side-effects of drugs with systemic symptoms: does a DRESS syndrome really exist?. Br J Dermatol.

[4] Lipowicz S., Sekula P., Ingen-Housz-Oro S. (2013). Prognosis of generalized bullous fixed drug eruption: comparison with Stevens-Johnson syndrome and toxic epidermal necrolysis. Br J Dermatol.

[5] Klaewsongkram J., Sukasem C., Thantiworasit P., ThaiSCAR study group (2019). Analysis of HLA-B allelic variation and IFN-γ ELISpot responses in patients with severe cutaneous adverse reactions associated with drugs. J Allergy Clin Immunol Pract.

